# Identifying sensitive windows of airborne lead exposure associated with behavioral outcomes at age 12

**DOI:** 10.1097/EE9.0000000000000144

**Published:** 2021-03-16

**Authors:** Erika Rasnick, Patrick H. Ryan, A. John Bailer, Thomas Fisher, Patrick J. Parsons, Kimberly Yolton, Nicholas C. Newman, Bruce P. Lanphear, Cole Brokamp

**Affiliations:** a Department of Statistics, Miami University, Oxford; b Division of Biostatistics and Epidemiology, Cincinnati Children’s Hospital Medical Center; c Department of Pediatrics, University of Cincinnati, Cincinnati, Ohio; d Division of Environmental Health Sciences, Wadsworth Center, New York State Department of Health, Albany; e Department of Environmental Health Sciences, School of Public Health, University at Albany, Rensselaer, New York; f Division of General and Community Pediatrics, Cincinnati Children’s Hospital Medical Center; g Department of Environmental and Public Health Sciences, University of Cincinnati, Cincinnati, Ohio; h Faculty of Health Sciences, Simon Fraser University, Burnaby, BC, Canada

**Keywords:** Air pollution, Child behavior, Distributed lag model

## Abstract

Despite the precipitous decline of airborne lead concentrations following the removal of lead in gasoline, lead is still detectable in ambient air in most urban areas. Few studies, however, have examined the health effects of contemporary airborne lead concentrations in children.

**Methods:**

We estimated monthly air lead exposure among 263 children (Cincinnati Childhood Allergy and Air Pollution Study; Cincinnati, OH; 2001–2005) using temporally scaled predictions from a validated land use model and assessed neurobehavioral outcomes at age 12 years using the parent-completed Behavioral Assessment System for Children, 2nd edition. We used distributed lag models to estimate the effect of airborne lead exposure on behavioral outcomes while adjusting for potential confounding by maternal education, community-level deprivation, blood lead concentrations, greenspace, and traffic related air pollution.

**Results:**

We identified sensitive windows during mid- and late childhood for increased anxiety and atypicality scores, whereas sensitive windows for increased aggression and attention problems were identified immediately following birth. The strongest effect was at age 12, where a 1 ng/m^3^ increase in airborne lead exposure was associated with a 3.1-point (95% confidence interval: 0.4, 5.7) increase in anxiety scores. No sensitive windows were identified for depression, somatization, conduct problems, hyperactivity, or withdrawal behaviors.

**Conclusions:**

We observed associations between exposure to airborne lead concentrations and poor behavioral outcomes at concentrations 10 times lower than the National Ambient Air Quality Standards set by the US Environmental Protection Agency.

## Abstract 2

Supplemental Digital Content is available in the text.

**What this study adds**

In this article, we show that exposure to very low concentrations of airborne lead during childhood is associated with poor behavioral outcomes at age 12. Controlling for potential confounders, we identified sensitive windows of exposure to airborne lead in mid- and late childhood for increased anxiety and atypicality scores and sensitive windows for increased aggression and attention problems immediately following birth. We believe that this manuscript is appropriate for publication by *Environmental Epidemiology* given the novelty of examining the impact of airborne lead exposure on child behavioral outcomes.

## Introduction

Decades of research support the association between blood lead concentrations and adverse neurobehavioral outcomes, even at levels below the current CDC reference level of 5 μg/dL.^1–8^ Elevated childhood blood lead levels have been associated with decreased reading and cognitive abilities, increased hyperactivity and impulsive behaviors, decreased gray matter volume in mood-regulating and decision-making regions of the adult brain, and higher rates of arrests for violent offences in adulthood.^9–13^

Exposure to lead occurs via four main routes: transplacental transfer, ingestion, inhalation, and remobilization of lead deposited in the bones^14,15^; the relative contribution of these sources to a child’s total lead exposure varies with age.^16^ When lead-based paint deteriorates, it contaminates house dust and soil.^14^ Ingestion can occur via hand-to-mouth contact or contaminated food or drink.^14^ Most of the lead is incorporated into bone and can leach back out into the body throughout life.^15^ Today, the highest concentrations of airborne lead, which are found in very small particle size, are due to nearby stationary sources such as industrial facilities and airports.^17–20^ Before the phase out of leaded gasoline began, however, leaded gasoline was the predominant source of airborne lead, accounting for over 50% of lead in blood.^19,21^ Between 1980 and 2014, ambient airborne lead concentrations decreased by 98%, shifting most lead exposure concerns to residential sources, such as lead-based paint and contaminated drinking water.^22^ However, detectable levels of ambient airborne lead still exist due to industrial emissions, transportation, and resuspended soil lead, and may travel directly to the brain due to their small particle size.^16,17,19,23,24^

Not only are children more susceptible to the effects of air pollution than adults, they are also more vulnerable to the neurotoxic effects of lead exposure during brain development.^25–28^ Assessing exposure over a time period during which a child is less susceptible to the effects of lead exposure can result in an underestimate of lead effects or absence of effects.^29^ Therefore, identifying windows of vulnerability is essential to fully understand the relationship between ambient lead exposure and neurobehavioral problems, and can provide etiological insights.

Despite the extensive research on health effects associated with blood lead concentrations, few studies have examined the impact of contemporary airborne lead concentrations on neurobehavioral outcomes. In this analysis, we estimated the impacts of exposures over specific time periods and tested for windows of increased vulnerability to ambient airborne lead exposure and behavioral problems in 12-year-old youth whose exposure to airborne lead never exceeded the United States Environmental Protection Agency (EPA) standard.

## Methods

### Study population

The Cincinnati Childhood Allergy and Air Pollution Study (CCAAPS) is a prospective birth cohort of children born from October 2001 to July 2003 in the Greater Cincinnati metropolitan region. Study eligibility required having a birth record address either farther than 1,500 m or closer than 400 m from a major highway and having at least one biologic parent with allergic sensitization verified by skin prick testing.^30,31^ The study was approved by the University of Cincinnati and Cincinnati Children’s Hospital Medical Center’s Institutional Review Boards (IRB approval #: 2012-1619), and caregivers provided written informed consent for their own and their child’s participation before enrollment.

### Airborne Pb exposure assessment

**Figure 1.**
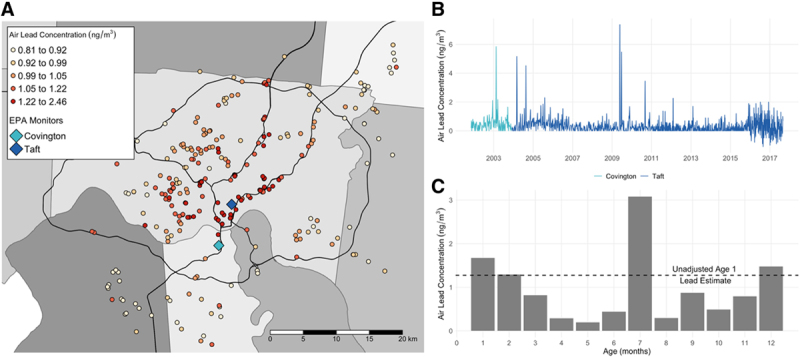
Spatiotemporal variation in airborne lead concentration in the Cincinnati area. A, Predicted ambient air lead concentrations from the LURF model before temporal scaling at CCAAPS children’s residential birth addresses. Specific address locations were jittered to maintain participant privacy. The different shades of gray represent different counties in the study area of the Greater Cincinnati, Ohio region in the United States and blue diamonds mark the location of EPA monitors in the study region used for temporal adjustment. B, Time series of airborne lead measurements recorded by EPA monitors in the study region from late 2001 to late 2017. These measurements were used to construct ratios and temporally scale predicted air lead from the LURF model. C, Temporally adjusted monthly air lead concentrations for the first year of life (bars) and unadjusted annual air lead concentration from the LURF model (dashed line) for a randomly selected CCAAPS child.

We used a previously validated exposure assessment model to estimate ambient concentrations of lead as a constituent of particulate matter of size 2.5 μm or smaller (PM_2.5_).^32^ Briefly, we created land use random forest (LURF) models based on ambient air sampling conducted in the study area at 24 different sites from 2001 to 2005. Land use predictors including land cover measurements, bus routes, greenspace, and population density were used to build a random forest model that had a cross validated accuracy pseudo-R^2^ of 0.89. The LURF model was used to estimate the airborne concentrations of lead at each child’s residence from birth through age 12, accounting for the length of residence in each home (Figure 1A).

To account for temporal variability in air lead concentrations, annual exposure estimates from the LURF model were scaled (monthly) using air quality system (AQS) data measured by EPA (Figure 1B).^33^ We constructed scaling factors using air lead measurements derived from PM_2.5_ measured every 3–6 days at a centrally located EPA monitoring station in the study area (monitor ID: 390610040). Air lead measurements before December 2003 were not available at this site, so data from a nearby site (monitor ID: 211170007) were substituted for this time period. To adjust for seasonal patterns of lead exposure, we constructed a scaling factor for each month of every participant’s life, from birth through age 12 years that was defined as the average monthly air lead level divided by the average air lead level from 2001 to 2005. Monthly exposure estimates were constructed by multiplying each scaling factor by the corresponding annual air lead estimate at the residential address from the LURF model (i.e., scaling factors for months 1–12 were multiplied by the age 1 time-weighted residential address estimate, scaling factors for months 13–24 by the age 2 estimate, and so on).^34^ For example, consider 9 October 2002 to be the birth date of a child of interest. The month 1 exposure estimate was obtained by adjusting the age 1 LURF model estimate by an adjustment factor:


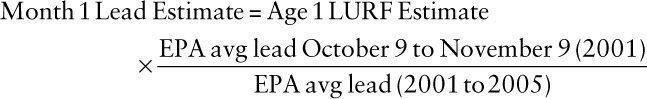


This was done for each month of each child’s life through their 12th birthday, for a total of 144 monthly estimates of airborne lead exposure specific to each study participant, increasing the temporal resolution and variability of estimated airborne lead exposure (Figure 1C). Annual average scaled airborne lead was uncorrelated with total PM_2.5_ exposure (age 1: *r* = −0.06, *P* = 0.29; age 12: *r* = −0.08, *P* = 0.22).

### Child behavioral assessment

The Behavioral Assessment System for Children, Second Edition, Parent Rating Scales (BASC-2) were completed by parents of CCAAPS participants at the 12-year visit. The BASC-2 was designed to assess the behavioral and emotional status of children and young adults in community and home settings, as well as aid in clinical diagnosis of behavioral and emotional disorders.^35^ BASC-2 subscale scores for internalizing behaviors (anxiety, depression, and somatization), externalizing behaviors (aggression, conduct problems, and hyperactivity), and others included in the behavioral symptoms index (attention problems, atypicality, and withdrawal) were selected as outcomes a priori based on existing evidence in the literature and biological mechanisms associated with air lead exposure. BASC-2 scores are reported as population normalized t-scores (with mean 50 and standard deviation 10), where scores above 59 are considered clinically at risk for problematic behaviors. BASC-2 measures of internal validity include the “Fake bad” (F) index, Consistency (C) index, and the Response Pattern (R) index. Observations were excluded from the analyses if the F-index score was >6, indicating that the parent rated the child overly negatively, the C-index was >17, indicating inconsistent responses to similar questions, or the R-index score was >125 or <66, suggesting parental inattention to the questionnaire.^35,36^

### Confounders

**Figure 2.**
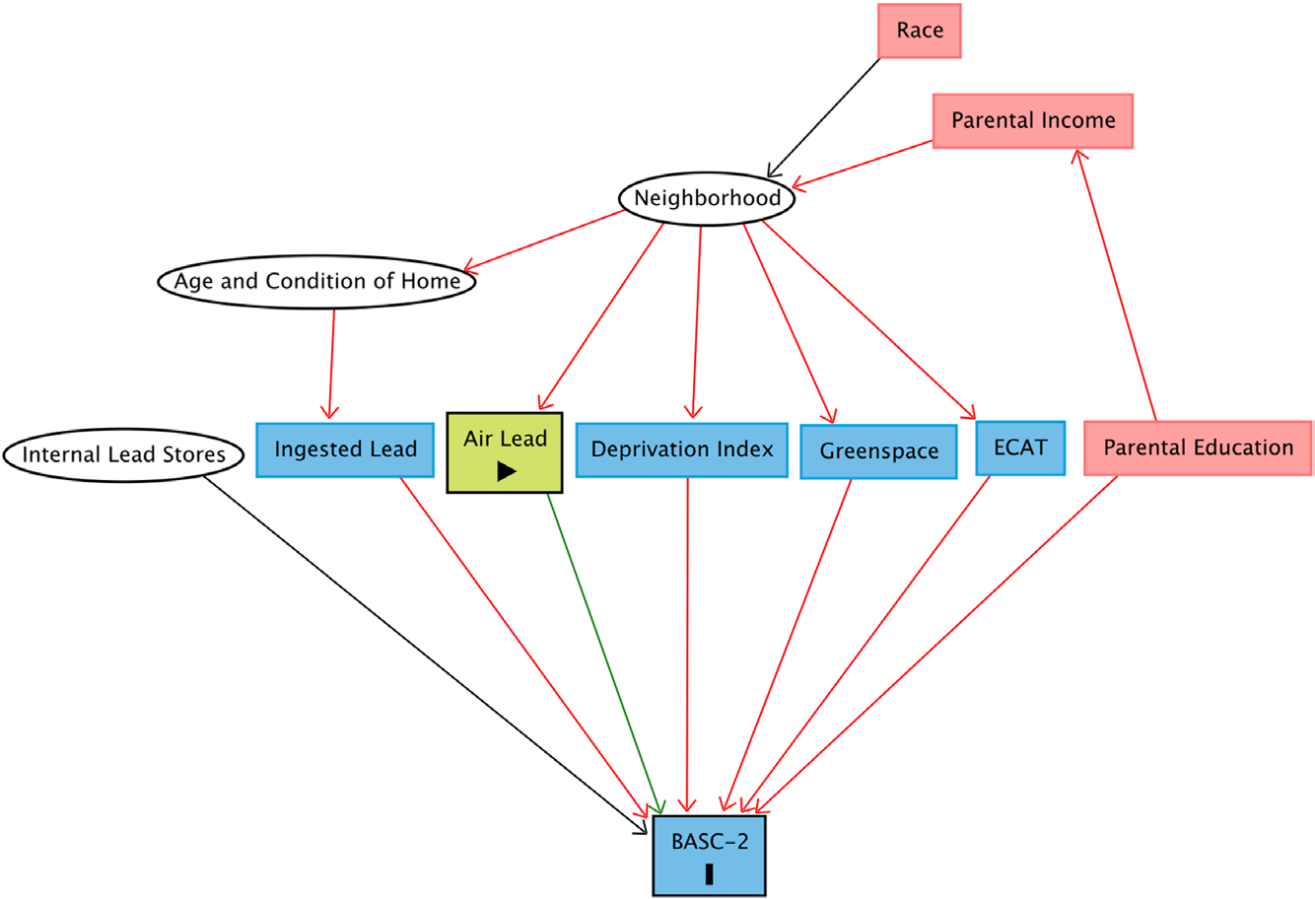
Directed acyclic graph quantifying the causal relationship between air lead exposure and BASC-2 scores at age 12, including potentially confounding pathways. Confounding exposures are red, competing exposures are blue, and unobserved exposures are in white circles.

We created a directed acyclic graph (DAG) based on currently available literature to identify pathways confounding the estimation of the causal relationship between air lead exposure and BASC-2 scores at age 12 (Figure 2). To estimate the direct effect of air lead on neurobehavioral problems, we used the DAG to identify a set of covariates for which to adjust in our statistical models. These included parental education, internal lead stores, neighborhood deprivation, nearby greenspace, and exposure to elemental carbon attributable to traffic (ECAT) as a marker of traffic-related air pollution exposure.^37^

Maternal education, a surrogate measure of the family’s socioeconomic status (SES) and child upbringing, was reported at the child’s 1-year follow-up visit (three levels: high school degree or less or trade school, some college, and college or graduate school). To account for internal lead stores, we adjusted for blood lead concentration measured at the child’s 12-year visit. Blood lead concentrations were measured via inductively coupled plasma-mass spectrometry using a method optimized for measuring very low background levels. The method is validated against National Institute of Standards and Technology Standard Reference Material 955c – Toxic Metals in Caprine Blood and has a limit of detection of 0.072 μg/dL.^38^ It is estimated that only one-third of lead measured in blood is due to inhaled lead,^39^ and in our cohort, average airborne lead exposure explained only 6% of the variation in blood lead, so we used blood lead level as a surrogate for internal lead stores to estimate the independent effect of airborne lead. To quantify neighborhood deprivation, we used a previously described index based on census tract-level data associated with each child’s geocoded residential address.^40^ Briefly, the deprivation index is the first principal component calculated via a principal components analysis of six socioeconomic census tract-level measures (fraction that graduated high school, fraction of households in poverty, median household income, fraction of population receiving public assisted income, fraction of houses that are vacant, and fraction of population with no health insurance coverage) from the 2015 American Community Survey. The index values range from 0 to 1, with higher values indicating greater deprivation.

An association between BASC-2 scores and greenspace, defined as open land with some type of vegetative cover, has been previously identified within our study population.^41^ Greenspace includes but is not limited to city parks, domestic gardens, nature strips, green roofs, cemeteries, and grounds of institutions such as hospitals and universities. Briefly, digital imagery numbers from satellite-derived normalized difference vegetation index (NDVI) images collected in June of 2010 were converted to surface reflectance values. Values within 400 m of each residential address were then averaged to obtain the NDVI value at that address. NDVI values range from −1 to 1, with higher values representing more greenspace.^41^ An association between ECAT and BASC-2 scores has also been previously identified in our study population.^36^

Measurements of residential greenspace, community deprivation, and ECAT were estimated based on residential addresses provided at the age 1-, 2-, 3-, 4-, 7-, and 12-year visits. We included an average of the six estimates in our models to represent the confounding effects of each of these variables.

### Statistical modeling

To identify sensitive developmental windows of exposure to air lead effects on BASC-2 scores, we used a distributed lag model (DLM) framework. In general, DLMs predict current values of a response based on current and past (“lagged”) values of a predictor.^42^ This is done by accounting for the relationship between the exposure and outcome while also accounting for time as a third dimension.^43,44^ DLMs have the flexibility to incorporate different functional forms to describe the time dimension and can be extended to distributed lag nonlinear models (DLNMs) to model nonlinear associations between the exposure and response. Since this is one of the first studies to estimate the relationship between airborne lead and behavioral outcomes, we chose to model a linear dose-response relationship between air lead and BASC-2 outcomes because we did not have any information on the shape of the dose–response relationship. An investigation of residuals from the model fit did not suggest any violations of the normality assumption. Exposures in discrete monthly intervals from birth to age 12 were modeled with regression coefficients associated with each time interval. Given the fit of these models, the relative importance of each exposure window can be assessed.

Specifically, we fit the linear DLM


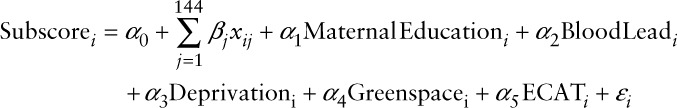


where 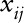 is the estimated air lead level in each month *j* of life for each subject *i*.

Without additional structure on the beta terms, this is a multiple linear regression model. However, contiguous exposures in time are often correlated due to temporal patterns in air pollution. Also, we constructed the 144 monthly exposure estimates per child as functions of their yearly exposure estimates, so exposures within one year are inherently related. To avoid unstable month-specific effect estimates due to multicollinearity, we fit a constrained DLM that assumes the effects are a smooth function of the month *j*, such that 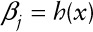.^29,43^ We modeled the smooth function 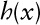 using penalized splines.^45^ A knot was placed at every lag, and a cubic regression penalty was applied, allowing for data-driven model selection through knot placement.

Each model fit resulted in estimated coefficients 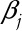 associated with a 1 ng/m^3^ increase in airborne lead exposure during each month of life *j* and a measure of effective degrees of freedom (edf) that describes the degree of smoothness and model complexity. A sensitive window was defined as any month(s) in which the 95% confidence interval for the coefficient excluded zero, and a higher edf indicated a more complex model shape (i.e., lower edf is closer to a linear fit and higher edf indicates more nonlinearity). To avoid overfitting, if an edf >20 was selected, we refit the model restricting to a maximum of 20 equally spaced knots. We also fit unadjusted DLMs to determine the degree of residual confounding.

### Statistical computing

Data analyses were completed in *R* (R Core Team; Vienna, Austria), specifically using the *dlnm* package to fit the DLMs.^43,46^

## Results

### Cohort characteristics

A total of 344 children completed the 12-year study visit and had BASC-2 data available. Of these, we excluded 55 children due to missing covariate information, 26 children with missing monthly airborne lead estimates (due to missing address data) and 8 children who had an invalid BASC-2 based on the publisher’s validity indices, resulting in a total of 263 children in the analysis. For each covariate, we found no significant differences between the group of 263 included children and the group of excluded children with the available covariate information (e.g., one of the 81 excluded participants was missing data for deprivation index, so the group of 80 excluded deprivation indices was compared to the group of 263 included deprivation indices). The number of values used to calculate averages for each excluded group is noted to the right of the average in Table 1. Children were exposed to median monthly air lead levels of 0.51 ng/m^3^ (range 0–10.8 ng/m^3^) (Table 1).

**Table 1.** Descriptive statistics of the CCAAPS cohort (mean ± SD unless otherwise noted)

	Included	Excluded	
N	263	81	
Female	122 (46%)	31 (38%)	
Maternal education			(N = 71)
High school or less	50 (19%)	22 (31%)	
Some college	77 (29%)	17 (24%)	
College/graduate school	136 (52%)	32 (45%)	
Greenspace (NDVI: [−1 to 1])	0.54 ± 0.08	0.55 ± 0.09	
Deprivation index [0 to 1]	0.41 ± 0.15	0.42 ± 0.16	(N = 80)
ECAT (μg/m^3^)	0.37 ± 0.10	0.39 ± 0.11	
Blood lead (μg/dL)	0.57 ± 0.37	0.53 ± 0.32	(N = 37)
Monthly air lead (ng/m^3^), median (IQR)	0.51 (0.56)	0.53 (0.59)	(N = 55)
BASC-2 scores			
Anxiety	52.1 ± 11.6	51.6 ± 12.1	
Depression	49.6 ± 9.9	50.4 ± 9.9	
Somatization	49.6 ± 11.2	48.8 ± 12.3	
Aggression	49.5 ± 8.7	48.1 ± 8.2	
Conduct problems	49.3 ± 8.8	48.0 ± 9.7	
Hyperactivity	50.7 ± 9.8	50.9 ± 11.0	
Attention problems	52.5 ± 9.8	52.5 ± 10.6	
Atypicality	50.0 ± 9.7	50.0 ± 9.6	
Withdrawal	50.0 ± 10.8	49.7 ± 11.4	

For categorical variables, percent of the total is displayed. For numeric variables, the values presented are the mean ± SD, unless otherwise noted. If covariate information was missing for children in the excluded group, the number of excluded children with available covariate information is noted to the right of each summary statistic. None of the averages for any covariates statistically differed for included and excluded participants.

### Identification of sensitive windows

**Figure 3.**
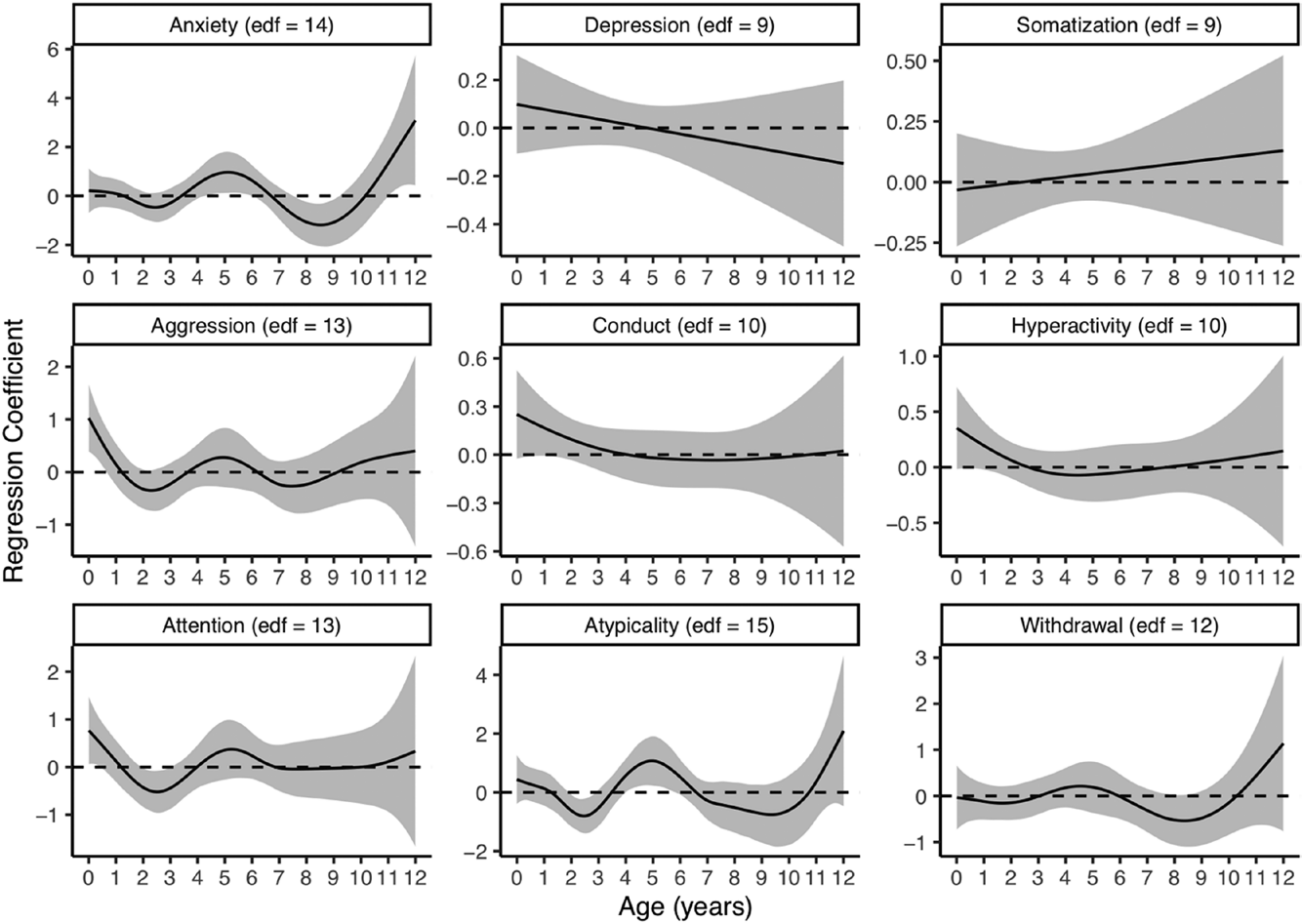
Associations between monthly airborne lead exposure levels from birth to age 12 and behavior scores from BASC-2 estimated using DLMs. Models were adjusted for maternal education level, blood lead at age 12, community deprivation, residential greenspace, and ECAT exposure. The *x* axis is the child’s age in years. The *y* axes (with differing scales for each outcome) represent the change in score associated with a 1 ng/m^3^ increase in airborne lead. Solid lines show the predicted change in score, and gray shading indicates the 95% CI. A sensitive window is identified for months where the estimated 95% CIs do not include zero.

Figure 3 displays the estimated β_j_-time relationship for the nine response scales along with their corresponding edf selected using our data-driven approach. Adjusting for maternal education, community deprivation, internal lead stores measured via blood lead concentration at age 12, greenspace exposure, and ECAT exposure, we observed possible associations during at least one sensitive window between airborne lead exposure and increased anxiety, aggression, attention problems, and atypicality scores (Figure 3). We identified sensitive windows from 4 years 4 months to approximately 5 years 11 months and from 11 years 1 month to approximately 12 years for increased anxiety scores. The effect peaked at age 12, where a 1 ng/m^3^ increase in airborne lead exposure was associated with a 3.1-point (95% CI: 0.4, 5.7) increase in anxiety scores (eTable S1; http://links.lww.com/EE/A124). Similarly, a probable sensitive window from 4 years 1 month to 5 years 8 months was identified for increased atypicality scores. The effect peaked at age 5, where a 1 ng/m^3^ increase in airborne lead exposure was associated with a 1.1-point (95% CI: 0.2, 2.0) increase in atypicality scores (eTable S1; http://links.lww.com/EE/A124). For aggression and attention problems, we identified sensitive windows early in life. Birth to 7 months was identified as a sensitive window for aggression, and birth to 5 months was identified as a sensitive window for attention problems. Both had relatively small effects that peaked at birth (aggression: 1.0; 95% CI: 0.4, 1.6; attention: 0.8; 95% CI: 0.1, 1.5) (eTable S1; http://links.lww.com/EE/A124). No sensitive windows were identified for increased depression, somatization, conduct problems, hyperactivity, or withdrawal behaviors. In contrast to our hypothesis, we found windows of decreased risk that immediately followed windows of high risk for anxiety (age 7 years 7 months to 9 years 2 months), attention problems (age 2 years to 2 years 11 months), and atypicality (age 2 years to 2 years 10 months).

Unadjusted DLMs resulted in nearly identical sensitive windows for anxiety, aggression, attention, and atypicality (eTables S1 and S2; http://links.lww.com/EE/A124). Additionally, unadjusted models identified sensitive windows with relatively small effect sizes for conduct problems, hyperactivity, and withdrawal (eTable S2; http://links.lww.com/EE/A124). An unrealistically high number of knots were originally selected for the unadjusted conduct problems model. After restricting the conduct problems model to a maximum edf of 20, a much more parsimonious model was fit.

## Discussion

Although much is known about lead-associated neurobehavioral effects, to our knowledge this is the first study to identify direct associations between airborne lead exposure and harmful effects on neurobehavior independent of blood lead concentration. Of note, exposure to airborne lead concentrations was associated with increased behavioral problems at levels 10 times lower than the EPA National Ambient Air Quality Standard (NAAQS). Specifically, the maximum rolling 3-month average airborne lead concentration measured by the EPA in our study area was 11.2 ng/m^3^ compared with the EPA NAAQS of 150 ng/m^3^.^47^ Compared with this threshold and airborne lead levels before lead was removed from gasoline, current ambient lead concentrations seem extremely low but are still orders of magnitude higher today than in preindustrial times.^48^ Although our observed associations between airborne lead exposures and elevated BASC-2 scores may not be clinically relevant for an individual, the effect sizes observed at low exposures are likely to have large public health implications due to the size of the population that is exposed to airborne lead. For example, if airborne lead concentrations increased by 1 ng/m^3^ at age 12 years, therefore increasing anxiety scores by 3.1 points on average, approximately 67 of every 1000 children would move from not at risk to clinically at risk for anxiety based on BASC-2 criteria. Of note, 1 ng/m^3^ is a relatively small increase in airborne lead concentration and would still be well under the current NAAQS.

Few studies have examined the relationship between airborne lead and neurobehavioral outcomes independent of blood lead, but one neighborhood-level study found that 3 years of elevated airborne lead emission concentrations was associated with a predicted 1- to 3-point reduction in child IQ.^49^ Studies in mice have shown that air pollution may travel directly to the brain via olfactory epithelium and olfactory nerves or via transport along sensory nerves of the trachea and bronchi.^23,24,50^ This mechanism is plausible for lead due to the small size of airborne lead particles from industrial and vehicular sources.^17^ Furthermore, the divalent metal transporter DMT1 found in olfactory nerves can transport lead and other metals to the brain.^51,52^ Unlike ingested lead, inhaled lead is not subject to the body’s digestive system defense mechanisms, which means that this route of exposure may result in adverse health consequences at much lower concentrations. Our results and similar studies of manganese exposures support this hypothesis.^53^ Furthermore, animal models have shown that even picomolar levels of lead can stimulate certain Ca-dependent brain enzymes.^54^

This study has several strengths. First, we were able to estimate address-specific airborne lead concentrations using a validated LURF model. We then adjusted those estimates using airborne lead data from EPA monitoring sites in our study area to achieve monthly temporal resolution. Second, we were able to estimate the relationship between airborne lead exposure and neurobehavior independent of lead from other sources of exposure by adjusting for blood lead concentration. Finally, we used a data-driven statistical method to identify patterns of association with respect to timing of exposure, rather than choosing time windows a priori over which to assess exposure, which could result in underestimated or missed effects. Furthermore, we utilized penalized splines to model the lag relationship, which address the problem of choosing the appropriate degree of complexity of the DLM.^45^ Most published studies using DLMs employ unpenalized methods, choosing specific model structures and knot placements without exploring or justifying model specifications. Imposing these specifications potentially creates biased estimates of the effects at each lag. It also is important to note that current selection methods are not effective for traditional unpenalized DLMs and produce less efficient estimators, and it has been shown that penalized DLMs show improved inferential properties compared with the standard unpenalized version.^45^

We also acknowledge limitations of this study. Like other studies, we were not able to apportion blood lead to sources or pathways of exposure. However, we felt it was appropriate to use blood lead as a surrogate for internal lead stores based on the lack of correlation between air lead and blood lead or between blood lead and nearby sources of lead. Omitting blood lead concentration as a covariate in our models did not meaningfully change our results, but these findings still highlight the need for further research and better methods for determining the impact of lead from different exposure pathways and sources. Because blood lead samples were only collected at the age 12 visit, we were unable to adjust for blood lead concentrations over time. Here, we considered blood lead a cumulative measure of lifetime exposure to noninhaled lead. Bone lead is a more accurate measure of cumulative exposure, but because lead stored in the bones is leached into the bloodstream throughout life, blood lead is in equilibrium with bone lead stores.^55^ Therefore, in the absence of repeated blood lead concentrations or a measure of bone lead, we used blood lead as a surrogate measure of cumulative exposure. This required us to assume that lead ingestion was either trivial at the time of the blood sampling or represented chronic, low-level exposure, which is likely in young adolescents.^25^

We were not able to assess prenatal exposures and their relationship with neurobehavior because we did not have prenatal residential address histories nor measures of airborne lead concentrations in our study area prior to November 2001. Furthermore, concentrations of airborne lead as a component of PM_2.5_ were correlated with concentrations of other metals that make up PM_2.5_, which could be confounding our results.^32^

Similar to other studies that used DLMs to examine early life air pollution exposures and health outcomes in children, we identified some windows of exposure that appear to be protective, such as the association between air lead from age 7 years 7 months to 9 years 2 months with decreased anxiety scores (Figure 3).^56–59^ There is no known biological mechanism that would explain why airborne lead exposure during any period of development would improve anxiety in children at age 12. These protective windows could be, in part, explained by the harvesting hypothesis, which assumes that a portion of the initial risk is discounted by a decrease in the susceptible pool after an event.^60,61^ In other words, if airborne lead caused problematic changes among children that were susceptible to its effects (i.e., “high risk” children) and had little effect on healthy children, then further exposure on subsequent days would not cause additional problematic changes in the high-risk children who were already affected. Fewer children would then be affected in the subsequent period because the effects of airborne lead are applied to a smaller at-risk pool.^61^ This paradox is an artifact of the counterfactual condition associated with the DLM backward perspective.^60^ Essentially, the association of the exposure and outcome over a specific lag period is compared with the association at a constant exposure. When the susceptible pool is depleted, as previously discussed, the observed population becomes “healthier” than the counterfactual population, which leads to apparent associations in the opposite direction than what is expected.^60^

Future studies could utilize model validation techniques to address the choice of function used to model the exposure–response relationship. Other studies have found evidence of a “supralinear” dose–response relationship for environmental toxicants such as lead, in which risk of negative health effects increased at a much greater rate at low levels of exposure compared with higher levels of exposure, and DLMs could be extended to DLNMs to explore these possible nonlinear dose–response relationships.^9,43,62,63^ Last, we used measures averaged over time to adjust for residential greenspace, ECAT, and community deprivation, but their associations with neurobehavioral outcomes could also vary in time and could be modeled using DLMs.

In conclusion, we identified potentially sensitive windows of exposure to exceedingly low concentrations of airborne lead and behavioral problem scores. This study is consistent with adverse consequences observed at very low blood lead concentrations. Due to the exploratory nature of identifying sensitive exposure windows and the multiplicity of comparisons assessed, follow-up studies in different cohorts are needed to replicate our findings and investigate the underlying mechanisms.

## Conflict of interest statement

The authors declare that they have no conflicts of interest with regard to the content of this report.

## ACKNOWLEDGMENTS

We thank the CCAAPS participating families, recruitment team, and clinical staff.

## Supplementary Material


