# Supplementary material for: Identifying sensitive windows of airborne lead exposure associated with behavioral outcomes at age 12
Source: Environ Epidemiol. 2021 Mar 16;5(2):e144. doi: 10.1097/EE9.0000000000000144 (PMC8043737; doi:10.1097/EE9.0000000000000144)
Supplement: Supplementary file 1 [file ee9-5-e144-s001.docx]

Supplementary Information for:

Identifying Sensitive Windows of Airborne Lead Exposure Associated with Behavioral Outcomes at Age 12

Erika Rasnick^1,2^, Patrick H. Ryan^3,2^, A. John Bailer^1^, Thomas Fisher^1^, Patrick J. Parsons^4,5^, Kimberly Yolton^3,6^, Nicholas C. Newman^3,6,7^, Bruce P. Lanphear^8^, Cole Brokamp^3,2^

^1^ Department of Statistics, Miami University; Oxford, OH, USA

^2^ Division of Biostatistics and Epidemiology, Cincinnati Children’s Hospital Medical Center; Cincinnati, OH, USA

^3^ Department of Pediatrics, University of Cincinnati; Cincinnati, OH, USA

^4^ Division of Environmental Health Sciences, Wadsworth Center, New York State Dept. of Health; Albany, NY, USA

^5^ Department of Environmental Health Sciences, School of Public Health, University at Albany, Rensselaer, NY, USA

^6^ Division of General and Community Pediatrics, Cincinnati Children’s Hospital Medical Center; Cincinnati, OH, USA

^7^ Department of Environmental and Public Health Sciences, University of Cincinnati; Cincinnati, OH, USA

^8^ Faculty of Health Sciences, Simon Fraser University; Burnaby, BC, Canada

Corresponding author: Erika Rasnick

[erika.rasnick@cchmc.org](mailto:erika.rasnick@cchmc.org)

3333 Burnet Avenue, Cincinnati, Ohio 45229

(513)517-7030

Table S1. Summary of Sensitive Windows Identified Using Adjusted DLMs

| **Outcome** | **Sensitive window(s)** | **Lag at peak effect** | **Peak effect estimate (μg/m^3^)** | **Peak effect 95% confidence interval (μg/m^3^)** |
| --- | --- | --- | --- | --- |
| Anxiety | 4y 4m – 5y 11m  7y 7m – 9y 2m  11y 1m – 12y | 12y | 3.1 | (0.4, 5.7) |
| Aggression | birth – 7m | birth | 1.0 | (0.4, 1.6) |
| Attention | birth – 5m  2y – 2y 11m | birth | 0.8 | (0.1, 1.5) |
| Atypicality | 2y – 2y 10m  4y 1m – 5y 8m | 5y | 1.1 | (0.2, 2.0) |

Table S2. Summary of Sensitive Windows Identified Using Unadjusted DLMs

| **Outcome** | **Sensitive window(s)** | **Lag at peak effect** | **Peak effect estimate (μg/m^3^)** | **Peak effect 95% confidence interval (μg/m^3^)** |
| --- | --- | --- | --- | --- |
| Anxiety | 4y 8m – 5y 6m  7y 4m – 9y 2m  11y 1m – 12 y | 12y | 2.9 | (0.3, 5.4) |
| Aggression | birth – 8m | birth | 1.0 | (0.4, 1.7) |
| Conduct | birth – 1y 9m | birth | 0.3 | (0.1, 0.6) |
| Hyperactivity | 1m – 8m | 1m | 0.3 | (0.0, 0.7) |
| Attention | birth – 7m  2y 2m – 2y 8m | birth | 0.9 | (0.2, 1.6) |
| Atypicality | 2y 1m – 2y 10m  4y 3m – 5y 7m  11y 7m – 12y | 12y | 2.8 | (0.0, 5.5) |
| Withdrawal | 7y 10m – 8y 3m | 7y 10m | -0.5 | (-1.0, 0.0) |
